# Soil stabilization linked to plant diversity and environmental context in coastal wetlands

**DOI:** 10.1111/jvs.12367

**Published:** 2016-01-04

**Authors:** Hilary Ford, Angus Garbutt, Cai Ladd, Jonathan Malarkey, Martin W. Skov

**Affiliations:** ^1^ School of Ocean SciencesBangor UniversityAngleseyLL59 5ABUK; ^2^ Centre for Ecology & HydrologyEnvironment Centre WalesBangorLL57 2UWUK

**Keywords:** Biodiversity–ecosystem–function, Ecosystem service, Erodibility, Erosion stabilization, Grassland, Plant species richness, Resilience, Root biomass, Salt marsh, Soil erosion

## Abstract

**Background:**

Plants play a pivotal role in soil stabilization, with above‐ground vegetation and roots combining to physically protect soil against erosion. It is possible that diverse plant communities boost root biomass, with knock‐on positive effects for soil stability, but these relationships are yet to be disentangled.

**Question:**

We hypothesize that soil erosion rates fall with increased plant species richness, and test explicitly how closely root biomass is associated with plant diversity.

**Methods:**

We tested this hypothesis in salt marsh grasslands, dynamic ecosystems with a key role in flood protection. Using step‐wise regression, the influences of biotic (e.g. plant diversity) and abiotic variables on root biomass and soil stability were determined for salt marshes with two contrasting soil types: erosion‐resistant clay (Essex, southeast UK) and erosion‐prone sand (Morecambe Bay, northwest UK). A total of 132 (30‐cm depth) cores of natural marsh were extracted and exposed to lateral erosion by water in a re‐circulating flume.

**Results:**

Soil erosion rates fell with increased plant species richness (*R* ^2^ = 0.55), when richness was modelled as a single explanatory variable, but was more important in erosion‐prone (*R* ^2^ = 0.44) than erosion‐resistant (*R* ^2^ = 0.18) regions. As plant species richness increased from two to nine species·m^−2^, the coefficient of variation in soil erosion rate decreased significantly (*R* ^2^ = 0.92). Plant species richness was a significant predictor of root biomass (*R* ^2^ = 0.22). Step‐wise regression showed that five key variables accounted for 80% of variation in soil erosion rate across regions. Clay‐silt fraction and soil carbon stock were linked to lower rates, contributing 24% and 31%, respectively, to variation in erosion rate. In regional analysis, abiotic factors declined in importance, with root biomass explaining 25% of variation. Plant diversity explained 12% of variation in the erosion‐prone sandy region.

**Conclusion:**

Our study indicates that soil stabilization and root biomass are positively associated with plant diversity. Diversity effects are more pronounced in biogeographical contexts where soils are erosion‐prone (sandy, low organic content), suggesting that the pervasive influence of biodiversity on environmental processes also applies to the ecosystem service of erosion protection.

**Nomenclature**
Stace (2010) for plants; Rodwell (2000) for plant communities 

## Introduction

Plants play a pivotal role in soil stabilization in many of the world's ecosystems, including grasslands, rivers and coastal wetlands (Durán Zuazo & Rodríguez Pleguezuelo 2008). Across these varied habitats, above‐ground shoots, laterally connected rhizomes or stolons and roots combine to protect against soil erosion by physically sheltering and fixing soils, offering resistance to rain, run‐off and attack by waves and currents (Gyssels et al. 2005). Root biomass, soil type and organic matter content are all important factors contributing towards variation in soil erosion rates (Gyssels & Poesen 2003; De Baets et al. 2006, 2007), with fine roots physically binding together soil particles, particularly clay or silt (Tengbeh 1993), and producing organic root exudates which support rhizosphere microbes that, in turn, excrete other soil cohesion elements (Reid & Goss 1981). The influence of individual biotic (e.g. root biomass) or abiotic (e.g. soil type) variables on soil stability are well quantified; however an understanding of how multiple factors, including plant biodiversity as a potential predictor of root biomass, combine to mitigate soil erodibility, is lacking.

Biodiversity affects ecosystem functioning (Isbell et al. 2011; Cardinale 2012; Gamfeldt et al. 2015), with diverse communities expected to become crucial to ecosystem service provision with emergent environmental change (Reich et al. 2012). High plant species richness may have a positive impact on ecosystem functions via: (1) ‘Functional complementarity’ where multi‐species communities perform better than any single species community, due to a high level of specialization between species, e.g. species specific rooting structures (Loreau et al. 2001); (2) The ‘Selection effect’, in which the specific functional traits of a dominant species may drive the response of plant mixtures (Hector et al. 2010); (3) The ‘Portfolio effect’, with species‐rich communities allowing asynchronous species fluctuations under conditions of environmental change, lowering system variability compared to single species communities (Doak et al. 1998; Naeem et al. 2012); or (4) ‘Facilitation’ or positive species interactions (Bruno et al. 2003), where one species makes the local environment more favourable for another, either directly (e.g. shading, nutrient uptake) or indirectly (e.g. deterring herbivores). Individual plant species vary in traits relating to erosion protection (Ghestem et al. 2014), with ‘ideal’ species possessing deep and extensive root systems with fast‐growing but strong roots that are resistant to decomposition (Stokes et al. 2009). Thus plant community composition, in concert with species diversity, should govern the local erodibility of soils, via root trait effects. There are indications that soil stability in alpine and steppe grasslands is positively correlated with plant species richness when species‐rich communities have higher root biomass and increased morphological complexity (Pohl et al. 2009; Liang‐Jun et al. 2013).

Landscapes under strong hydrological control, such as salt marshes, are ideal systems for studying the role of biology in resisting soil erosion, as they are often highly dynamic, with periods of erosion and expansion (Adam 1990; Gedan et al. 2011). Salt marsh expansion is often driven by small vegetated patches resisting erosion, encouraging sediment trapping and providing further opportunities for marsh expansion via a biophysical feedback loop (Langlois et al. 2003; Temmerman et al. 2005). Ultimately, the distribution and long‐term stability of salt marshes is controlled by these inter‐linked biotic and abiotic conditions (Van de Koppel et al. 2005; D'Alpaos 2011). Across salt marsh biogeographical regions, the abiotic factor of primary importance is soil grain size, with fine‐grained clay soils resisting erosion better than weaker sandy sediment (Van Eerdt 1985; Allen 1989). However, within regions controlled for soil type, biological factors are likely to become more prominent. Salt marsh plants play a vital role in coastal protection, and the importance of above‐ground vegetation in attenuating wave energy is well quantified (Möller et al. 1996, 1999). Salt marshes can reduce the height of storm waves by ~20%, with 60% of this due to vegetation (Möller et al. 2014). Even under conditions where surface vegetation was removed by intense wave action, the root network remained and the marsh surface successfully resisted erosion (Möller et al. 2014). Below‐ground vegetation enhances salt marsh soil stability, with higher root biomass and the presence of a finely distributed root network reducing soil erosion rates (Coops et al. 1996; Chen et al. 2012). Recent work has linked increased salt marsh biomass to plant species richness via niche complementarity and species selection (Sullivan et al. 2007; Stuedel et al. 2011); here we attempt to disentangle the relationship between plant community diversity, root biomass patterns and erosion protection via soil stabilization.

We examined whether soil stabilization was linked to elevated plant diversity in salt marshes, an ecosystem with a key role in shoreline protection. Marsh erodibility was studied in two geographical regions with contrasting soil properties to examine if the role of vegetation was context‐dependent: erosion‐prone Morecambe Bay (coarse‐grained, organically poor soil) and erosion‐resistant Essex (fine‐grained, organically rich soil). An erosion model was also constructed to assess the contribution of biodiversity, relative to other biotic and abiotic explanatory variables, to soil stability. The following three hypotheses were tested: (1) reduced soil erosion rate is associated with increased plant diversity; (2) root biomass is positively associated with plant diversity; and (3) plant diversity will contribute more to soil stability in regions with erosion‐prone than erosion‐resistant soils.

## Methods

### Site description and experimental design

Salt marsh sites (Table 1) were chosen to represent two contrasting soil types; clay soil in Essex (southeast Atlantic UK) and sandy soil from the greater Morecambe Bay area (northwest UK). All field sites were sampled in summer 2013 (Aug/Sept). Each site consisted of a rectangular area of salt marsh between 400 × 500 m to 1000 × 1000 m in size, dependent upon salt marsh length (parallel to shore) and width (perpendicular to shore), including part of the low‐, mid‐ and high‐marsh zones. Twenty‐two 1 × 1 m quadrats were randomly allocated to each site rectangle using R (R Foundation for Statistical Computing, Vienna, AT).

**Table 1:** Salt marsh site descriptions

Region	Site		Coordinates	Soil	Grazing
Essex	Abbotts Hall	AH	51° 47′ N, 0°52′ E	Clay	Brent geese, hares
	Fingringhoe Wick	FW	51°49′ N, 0°58′ E	Clay	Brent geese, hares
	Tillingham marsh	TM	51°41′ N, 0°56′ E	Clay	Hares
Morecambe Bay	Cartmel Sands	CS	54°10′ N, 3°0′ W	Sand	Sheep (~4–5 ha^−1^), pink‐footed geese
	West Plain	WP	54°9′ N, 2°58′ W	Sand	Sheep (<2 sheep ha^−1^)
	Warton Sands	WS	54°8′ N, 2°48′ W	Sand	Sheep (~4–5 ha^−1^), pink‐footed geese

Brent geese (*Branta bernicla*) and pink‐footed geese (*Anser brachyrhynchus*) are seasonal visitors only, over‐wintering on salt marshes.

### Soil erosion cores

**Figure 1:**
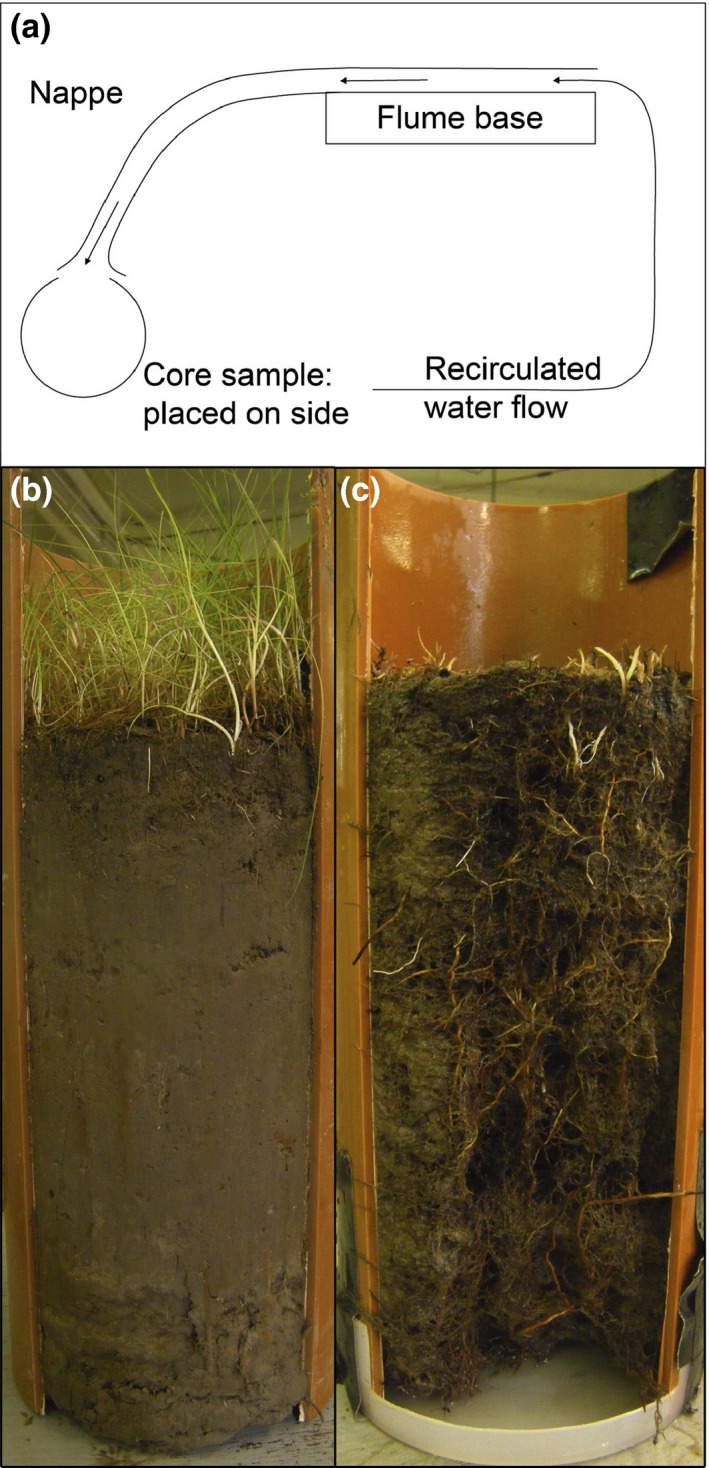
Re‐circulating flume set up (a) with example core prior to (b) and after (c) erosive treatment. Above‐ground vegetation has been removed in (c) but roots are clearly visible.

Prior to soil erosion measurements, one large cylindrical sediment core (16‐cm diameter, 30‐cm height) including above‐ground vegetation (additional 10 cm height) was collected from within each 1 × 1 m quadrat. Cores were bevelled on the lower edge and a serrated edge knife used to cut down into soil around the core perimeter to cut through large roots and ensure a smooth passage through the soil with minimal compaction. A 10‐cm wide slot was cut through the entire length of the vertical face of the core, and the exposed core surface placed horizontally under the nappe of a recirculating overshoot‐weir flume. Figure 1 shows a sketch of the flume and examples of the cores before and after the test. The stagnation pressure associated with flow being forced to change direction directly over the slot caused sediment to be eroded, representing side impact on the margin of a vegetated bank by waves and currents. The way the flume was used is analogous to a cohesive strength meter (Vardy et al. 2007) on a larger scale, except that rather than seeking to find the critical stagnation pressure that corresponds to the erosion threshold being passed, it is the cumulative loss of sediment mass that is being considered here. For each test the flume was run for 1.5 h for three different discharges, corresponding to three different stagnation pressures over the sample [0.5 h at low (61 Pa), 0.5 h at medium (146 Pa) and 0.5 h at high pressure (351 Pa)]. Sediment erosion rate was calculated from mass loss over 0.5 h at medium pressure and expressed as ‘% mass loss·min^−1^’. The low pressure was used to ensure full saturation of cores. Results of the high‐pressure condition are not shown here as some cores were completely destroyed. The coefficient of variation (CoV) of the erosion rate was also calculated for each species richness value (number of species·m^−2^).

### Vegetation characteristics

Above‐ground vegetation characteristics were measured from within each 1 × 1 m quadrat and from each large soil core, taken to observe sediment erosion rate in the laboratory. Percentage cover of each plant species within quadrats and cores was estimated by eye. Plant species richness was recorded as the number of species present per quadrat or per core. Shannon‐Wiener index [S‐W index (H')] was also calculated for each quadrat and core as a measure of plant species diversity (based on species cover). British national vegetation communities (NVC) were identified for each quadrat using Tablefit v1.1 (http://www.ceh.ac.uk/services/tablefit-and-tablcorn#tablcorn). Above‐ground dry vegetation biomass (60 °C, 72 h) was determined by cutting plants to ground level from a 50 × 25 cm area within each quadrat and from the total surface area of the core. Root dry biomass (60 °C, 72 h) was determined for three core depth sections: 0–10, 10–20 and 20–30 cm. Sections were extracted after cores were subjected to erosive tests (see ‘Soil erosion cores’) and roots were removed from sediment via washing. Collection of root biomass across a 0–30‐cm depth zone was considered sufficient to capture the majority of plant roots for most common salt marsh plant species.

### Soil characteristics

Elevation and *x*,* y* coordinates of each quadrat were measured to within ± 0.05 m (Leica GS08 GNSS system). Elevation was recorded in metres relative to Ordnance Datum Newlyn (ODN), converted to Chart Datum (CD) and presented relative to Mean High Water Neap (MHWN) as a proxy for tidal inundation. Soil samples, of ~10 g (fresh mass) from the top 10 cm, were taken from within each quadrat, diluted 1:2.5 by volume with deionized water and measured for electrical conductivity (EC) and pH (Jenway 4320 conductivity meter). EC was used as a proxy for salinity. Soil bulk density samples were taken using a stainless steel ring (3.1‐cm height, 7.5‐cm diameter) to vertically quantify three depth zones; 0–10, 10–20 and 20–30 cm, directly adjacent to the large soil erosion core, on the side of the core hole. Samples were dried (105 °C, 72 h) prior to calculation of bulk density. Soil moisture content was also calculated. The dried bulk density samples were ground and sub‐sampled to provide sediment for organic matter content and grain size analysis. Loss‐on‐ignition (375 °C, 16 h) was used to estimate organic matter content (Ball 1964). Soil carbon stock was calculated from bulk density with the conversion factor of soil carbon estimated as 0.55 of soil organic matter (http://countrysidesurvey.org.uk/sites/default/files/pdfs/reports2007/CS_UK_2007_TR9-revised.pdf). Prior to grain size analysis, any organic matter in ~3 g soil was digested using hydrogen peroxide. Soil was classified into 100 size fractions from 0.2–2000.0 μm (Malvern Particle Sizer 2000) and grouped according to the International Society of Soil Science (ISSS): clay = 0.2–2.0 μm; silt = 2–20 μm; fine sand = 20–200 μm; coarse sand = 200–2000 μm (2 mm).

### Statistical analysis

All analyses were carried out in R. To test for significant differences between the two regions (Essex, Morecambe bay) and six sites for vegetation, soil and erosion rate variables we employed linear models and used ANOVA output to assess for effects, followed by post‐hoc Tukey tests (http://multcomp.r-forge.r-project.org). Variables were logged where appropriate to normalize data and meet the assumptions of homogeneity of variance. Relationships between erosion rate and plant species richness were examined using pseudo *R* ^2^ output of linear mixed effects models (http://cran.r-project.org/package=nlme). Model selection was conducted by comparing Akaike information criterion (AIC) and qqnorm plots of each model fit. Classic model fit with a nested structure, ‘random = ~1 region/site/quadrat’ was compared to an exponential model, designed to control for spatial autocorrelation between quadrat observations based on the *x* and *y* coordinates of each quadrat. Step‐wise regressions ‘forwards and backwards’ were carried out in the ‘MASS’ package (Venables & Ripley 2002) on the results of a linear model [e.g. ‘lm (log (Erosion rate) ~ NVC + Plant species richness + Plant S‐W + Plant cover + Above‐ground biomass + Root biomass + Organic matter + Carbon stock + Clay‐silt fraction] to find out which combination of environmental factors best explained soil erosion rate (worked example) and root biomass. Predictor variables were only entered into the step‐wise regression if hierarchical partitioning (http://cran.r-project.org/package=hier.part) analysis assessed them to have ≥5% independent effects. Results of the step‐wise regression displayed a ‘final model’ selected by lowest AIC, usually with less variables than the ‘initial model’. From this model the individual contribution of each remaining environmental variable to the overall variation explained was calculated using the ‘lmg’ function of the ‘relaimpo’ package (Grömping 2006) using simple un‐weighted averages as recommended (see Appendix S1 for further detail on step‐wise regression predictor selection).

## Results

### Regional characterization

Plant species richness was higher in Morecambe Bay (~4–7 species·m^−2^) than Essex (~3–4 species·m^−2^). West Plain was the most species‐rich marsh, with 13 plants recorded from one quadrat. Plant S‐W index (H') was also higher for Morecambe Bay than Essex (Table 2). Vegetation core section results are presented in Appendix S2. Above‐ground biomass was significantly higher in Essex (~0.8 kg DW·m^−2^) than Morecambe Bay (~0.1–0.5 kg DW·m^−2^; Appendix S3). For total root biomass (0–30 cm) there was no significant regional difference. Despite this, Fingringhoe and West Plain had markedly higher root biomass (~5.5–8.0 kg DW·m^−2^) than the other salt marsh sites (~1–4 kg DW·m^−2^; Appendix S3). The ratio between above‐ground and root biomass was not consistent across regions.

**Table 2:** Site characteristics for six salt marshes within two regions, means per site are shown ± SD

	Essex			Morecambe Bay			*P*
	AH	FW	TM	CS	WP	WS	
Elevation (m) Relative to MHWN	1.09 ± 0.11 ab	1.21 ± 0.06 b	1.03 ± 0.09 a	2.33 ± 0.31 c	2.86 ± 0.10 d	2.88 ± 0.17 d	***
Vegetation ‐ Quadrat							
Plant Species Richness	4.7 ± 1.2 ab	4.4 ± 1.5 ab	3.8 ± 1.0 a	4.2 ± 1.4 a	7.3 ± 2.1 c	5.5 ± 1.0 b	***
S‐W Index (H')	0.97 ± 0.24 ab	0.98 ± 0.29 ab	0.74 ± 0.35 a	0.72 ± 0.45 a	1.23 ± 0.32 bc	1.22 ± 0.37 bc	**
Cover (%)	101 ± 12 a	104 ± 4 ac	95 ± 10 ab	87 ± 19 b	108 ± 11 c	104 ± 8 c	n.s.
Soil							
Electrical Conductivity (mS·cm^−1^)	25 ± 4 a	28 ± 7 b	22 ± 3 a	5 ± 4 c	3 ± 3 cd	3 ± 3 d	***
pH	6.9 ± 0.2 a	6.8 ± 0.3 a	7.4 ± 0.2 bc	7.5 ± 0.3 b	6.8 ± 0.6 a	7.3 ± 0.5 c	**
Moisture Content (%) 0–10 cm depth	58 ± 7 c	61 ± 9 c	46 ± 5 b	26 ± 11 a	39 ± 11 b	27 ± 11 a	***
Bulk Density (g·cm^−3^) 0–30 cm	0.56 ± 0.16 a	0.51 ± 0.15 a	0.83 ± 0.13 b	1.37 ± 0.14 e	1.09 ± 0.14 c	1.23 ± 0.12 d	***
Carbon Stock (t C·ha^−1^) 0–30 cm	113 ± 9 c	119 ± 12 c	90 ± 15 b	38 ± 18 a	93 ± 30 b	52 ± 15 a	***
Clay‐Silt Fraction (%)	93 ± 2 c	87 ± 7 c	90 ± 3 c	8 ± 4 a	17 ± 12 b	7 ± 4 a	***

AH, Abbotts Hall; FW, Fingringhoe Wick; TM, Tillingham marsh; CS, Cartmel Sands; WP, West Plain; WS, Warton Sands.

Letters denote significant site differences, final column significant regional differences.

Region *P <* *0.05, **P <* *0.01, ***P <* *0.001.

Essex marshes extended lower onto the intertidal shore; soils were saltier and with finer, more organically rich sediment than Morecambe Bay (Table 2; Appendix S3; see Appendix S4 for further detail). Essex soils were classified as ‘clay’ soils (>11% clay), Morecambe Bay as ‘sandy’ soils [% silt + (% clay × 2) < 30%] as in LandIS [2014 (http://www.landis.org.uk/)].

### Plant diversity and soil stabilization

**Figure 2:**
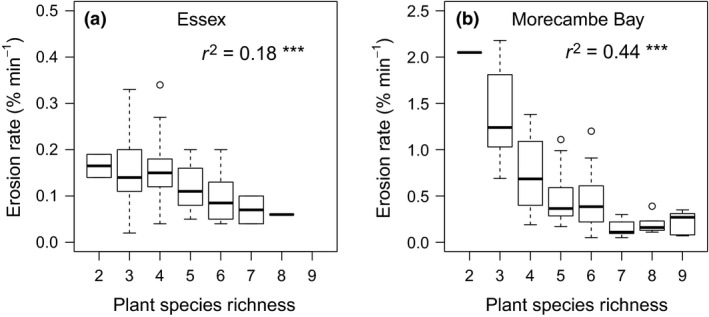
Results of mixed effects model with pseudo r^2^ showing proportion of variation in erosion rate explained by plant species richness per m^2^ in a) Essex and b) Morecambe Bay, *** P < 0.001. Thick bar = median, box = interquartile range, whiskers = full range, open circles = outliers.

The mean erosion rate was significantly higher for Morecambe Bay (0.3–1.0% mass loss·min^−1^) than Essex (<0.2% mass loss·min^−1^) for salt marsh soil cores (Appendix S5). Increased plant species richness, when modelled as a single explanatory factor, was associated with lower erosion rates for both regions combined (*R* ^2^ = 0.55, *F *=* *18.62, *P *<* *0.001). Plant species richness explained more variation in erosion rates in Morecambe Bay (*R* ^2^ = 0.44, *F *=* *19.11, *P *<* *0.001; Fig. 2b) than Essex (*R* ^2^ = 0.18, *F *=* *12.07, *P *<* *0.001; Fig. 2a). Reduction in between‐sample variation (CoV) in soil erosion rate with higher plant species richness was also apparent (*R* ^2^ = 0.92, *F *=* *79.32, *P *<* *0.001).

**Figure 3:**
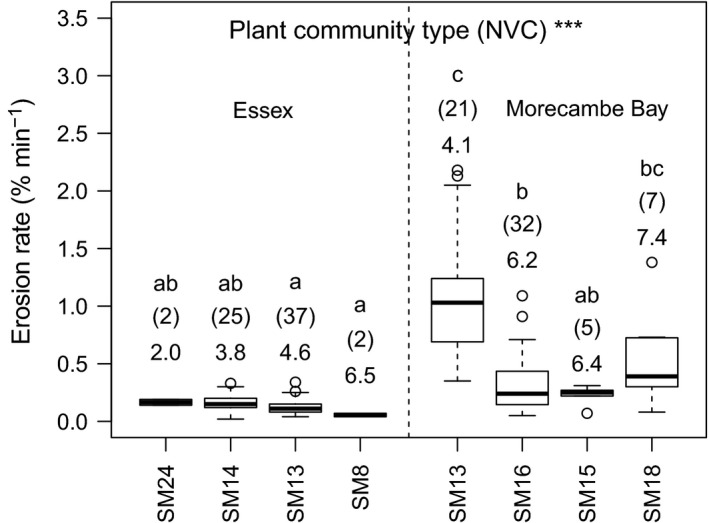
Relationship between soil erosion rate and plant community type, within region, ***P < 0.001. NVC type with dominant species: SM24 = *Elytrigia atherica*, SM14 = *Atriplex portulacoides*, SM13 = *Puccinellia maritima*, SM16 = *Juncus gerardii*, SM15 = *Juncus maritimus* / *Triglochin maritima*, SM18 = *J*. *maritimus*. Italicised letters denote significant differences *P* < 0.05. Number immediately above each bar denotes mean species richness for each community type, n in brackets. Thick bar = median, box = interquartile range, whiskers = full range, open circles = outliers.

Plant diversity indices were positively correlated to root biomass (Plant species richness, *r*_s_ = 0.63, *P *<* *0.001; Plant S‐W, *r*_s_ = 0.54, *P *<* *0.001) and MHWN (plant species richness *r*_s_ = 0.44, *P *<* *0.001; Plant S‐W, *r*_s_ = 0.38, *P *<* *0.001), with plant species richness increasing in line with marsh elevation gradients. Relationships between plant diversity indices and soil type (based on clay‐silt fraction, bulk density) were generally lower (<20%) and not significant. Plant community types (NVC), characteristic of each region, exhibited significantly different sediment erosion rates (*F *=* *23.58, *P *<* *0.001; Fig. 3). *Puccinellia maritima* communities from Essex had significantly lower erosion rates than the same community type in Morecambe Bay (*P *<* *0.001). Within Morecambe Bay, *Juncus gerardii* and *Juncus maritimus* communities were both indicative of lower erosion rates than the *P. maritima* community (*P *<* *0.05).

### Influence of plant diversity on root biomass

Step‐wise regression of all potential root biomass predictors for Essex and Morecambe Bay combined produced a final model that explained 42% of the overall variation in root biomass (Table 3). Plant species richness and plant cover were the most important explanatory variables for both the combined region model and the Morecambe Bay model, accounting for ~20% and 12–18% of root biomass variation, respectively. For Essex, plant species richness accounted for 32% of the variation, with elevation above MHWN an important secondary predictor (Table 3).

**Table 3:** Root biomass predictor variables identified by best fit models (step‐wise regression) for Essex and Morecambe Bay salt marshes (combined and regional)

Model variables	Estimate	SE	*t*‐Value	*P* value	*R* ^2^
Best model fit: both regions (AIC = 276.66, F = 21.19, *df* = 4, 115, *P* < 0.001 ***, *R* ^2^ = 0.42)					
Plant Species Richness	1.043	0.217	4.801	4.79 × 10^−6^ ***	0.22
Plant Cover (%)	0.064	0.027	2.329	0.022*	0.12
Clay‐Silt Fraction (%)	0.045	0.027	1.670	0.098 n.s.	0.03
MHWN	2.011	1.415	1.421	0.158 n.s.	0.05
Best Model Fit: Essex (AIC= 108.16, F = 31.6, *df* = 2, 58, *P* < 0.001 ***, *R* ^2^ = 0.52)					
Plant Species Richness	1.440	0.233	6.188	6.65 × 10^−8^ ***	0.32
MHWN	12.99	2.670	4.865	9.15 × 10^−6^ ***	0.20
Best Model Fit: Morecambe Bay (AIC = 151.68, F = 14.86, *df* = 3, 55, *P* < 0.001 ***, *R* ^2^ = 0.45)					
Plant Species Richness	0.800	0.343	2.333	0.023*	0.19
Plant Cover (%)	0.097	0.038	2.563	0.013*	0.18
Clay‐Silt Fraction (%)	0.083	0.058	1.413	0.163 n.s.	0.08

*P *<* *0.05*, *P *<* *0.01**, *P *<* *0.001***, n.s.  = *P *>* *0.05.

### Relative importance of biotic and abiotic factors to soil stabilization

Step‐wise regression, of all potential erosion rate predictors, for both regions combined, produced a final erosion model of five measured variables explaining 80% of the overall variation in erosion rate (Table 4). Plant S‐W diversity index, plant cover and root biomass in turn accounted for 4, 8 and 13% of overall variation. The Clay‐silt fraction and soil carbon stock explained 24% and 31%, respectively. When the data set was split by region, and best fit models compared (Table 4), the most noticeable differences were: (1) measured variables in the Morecambe model explained much more of the statistical variation in erosion rate (78%) than the Essex model (46%); (2) grain size (clay‐silt fraction) was not selected as a predictor variable within regions; (3) plant diversity (S‐W index) and plant cover were better predictors of erosion rate in Morecambe Bay (12 and 18%) than Essex (not selected). Note that plant diversity indices were selected (via step‐wise regression) as a final model component, above plant community type (NVC) in both combined and regional models.

**Table 4:** Predictor variables of soil erosion rate identified from best fit models (step‐wise regression) for Essex and Morecambe Bay salt marshes (combined and regional)

Model variables	Estimate	SE	*t*‐Value	*P* value	*R* ^2^
Best Model Fit: Both Regions (AIC= ‒−181.42, F = 88.1, *df* = 5, 114, *P* < 0.001 ***, *R* ^2^ = 0.80)					
Plant S‐W Index (H')	−0.335	0.127	−2.637	0.009**	0.04
Plant Cover (%)	−0.012	0.004	−2.950	0.004**	0.08
Root Biomass (kg DW·m^−2^)‡	−0.072	0.014	−5.129	1.21 × 10^−6^ ***	0.13
Clay‐Silt Fraction (%)	−0.011	0.002	−6.063	1.78 × 10^−8^ ***	0.24
Carbon Stock (t C·ha^−1^)‡	−0.009	0.002	−4.073	8.59 × 10^−5^ ***	0.31
Best Model Fit: Essex (AIC= ‒−100.17, F = 24.6, *df* = 2, 58, *P* < 0.001 ***, *R* ^2^ = 0.46)					
Root Biomass (kg DW·m^−2^)‡	−0.072994	0.017309	−4.217	8.78 × 10^−05^ ***	0.24
Carbon Stock (t C·ha^−1^)‡	−0.013348	0.003303	−4.041	0.000159***	0.22
Best Model fit: Morecambe Bay (AIC = −‒83.72, F = 48.7, *df* = 4, 54, *P* < 0.001 ***, *R* ^2^ = 0.78)					
Plant S‐W Index (H')	−0.362457	0.177	−2.045	0.046*	0.12
Plant Cover (%)	−0.014597	0.006	−2.652	0.010*	0.18
Root Biomass (kg DW·m^−2^)†	−0.085568	0.020	−4.287	7.52 × 10^−5^ ***	0.27
Carbon Stock (t C·ha^−1^)†	−0.008092	0.003	−2.961	0.005**	0.21

A negative relationship with soil erosion rate infers a positive relationship with physical soil stability.

^†^Based on pooled 0–30 cm soil depth.

^‡^Based on pooled 0–30 cm soil depth.

*P *<* *0.05*, *P *<* *0.01**, *P *<* *0.001***.

## Discussion

### Plant diversity and soil stabilization

This study examined whether soil stabilization was associated with plant diversity. We found that soil erosion rate reduced concurrently with increased plant species richness, implying that biodiversity could enhance erosion protection by plants in coastal wetlands. While the study did not experimentally manipulate biodiversity and therefore cannot firmly establish a causative relationship of plant diversity on soil stabilization, it did factor out a range of other plausible biological and environmental explanatory variables of soil erodibility, and found biodiversity remained a significant explanatory variable of variation in soil erosion. The association between soil stabilization and plant diversity was much stronger in erosion‐prone sandy soils than erosion‐resistant clay soils, implying biodiversity might be particularly important in settings where erosion risk is inherently higher.

As small‐scale plant species richness increased, the between‐sample variation in the rate of soil erosion declined strikingly, indicating that diverse plant communities can limit variability in ecosystem processes, as shown in manipulated grassland plot experiments (Tilman et al. 1997; Stuedel et al. 2011). Our study demonstrates this biodiversity–variability effect in a naturally occurring plant community, but the mechanisms behind this soil stabilization effect remain uncertain. The naturally species‐rich plant communities sampled within this study are also often functionally diverse, leading to enhanced root biomass and differing root growth strategies via functional complementarity (Loreau et al. 2001). In contrast, species‐poor communities range from excellent to minimal soil stabilizing properties, dependent on their specific growth type (De Baets et al. 2009). Species‐rich plant communities may be able to compensate more for changing environmental conditions than species‐poor communities and therefore maintain ecosystem functions (Loreau et al. 2001) such as erosion stabilization.

The respective effects on soil stabilization from plant diversity and root biomass may prove difficult to disentangle, as one of the ways in which diversity is expected to mediate erosion is via enhanced root biomass. Here we found that plant species richness and plant cover were the most important explanatory variables of root biomass. Plant diversity and root biomass also had a tendency to increase with marsh elevation, potentially as salt stress reduces up the shore. Bouma et al. (2001) found an adaptive relationship between root branching structure and elevation in several salt marsh species with annual dicots (e.g. *Salicornia*), with dichotomous branching at high elevations that allowed for rapid acquisition of nutrients in a competitive and nutrient‐limited environment. These dichotomous or ‘ever splitting’ rooting systems might be better at erosion control than slow‐growing herringbone root structures common in some salt marsh grasses and at lower elevations. Erosion protection by roots might, therefore, be strongest furthest away from the salt marsh edge where it is least required.

Soil erodibility was associated with plant community type, particularly in erosion‐prone sandy soils; with cores from *P. maritima* communities eroding more than twice as fast as those from *J. gerardii* communities. Here we found that the salt marsh grass *P. maritima,* a stolon‐producing perennial with fibrous roots, occurred predominantly as a near monoculture, whereas the tufted graminoid rush *J. gerardii* commonly grew alongside the grass *F. rubra* and various forbs. *J. gerardii* communities exhibit a range of rooting structures; *J. gerardii* itself has extensive laterally‐creeping rhizomes with thick anchors and many shallow fine roots, *F. rubra* is a perennial with deep roots reaching down to 40 cm (Brown et al. 2010) and a commonly co‐occurring forb, white clover (*Trifolium repens*), is stoloniferous with nitrogen fixing nodules (http://www.fao.org/ag/agp/agpc/doc/gbase/data/Pf000350.htm). Thus, the functionally diverse *J. gerardii* community exhibited a wide variety of different rooting structures and depths (Minden et al. 2012), enhancing soil stability via niche complementarity (Sullivan et al. 2007; Stuedel et al. 2011). Where the same plant community was found in both geographical study regions, erosion rates were far higher in the erosion‐prone sand‐dominated region, compared to the erosion‐resistant clay‐dominated region, highlighting the over‐arching importance of soil type on erosion mitigation.

### Erosion model

Experimental studies to date have rarely explained the complex relationship between biological and environmental drivers that in turn determine ecosystem function (Maestre et al. 2012; Midgley 2012). Here we present an erosion model that includes biodiversity and explains 80% of the variation in sediment erosion rate in salt marsh grasslands. Biotic factors, primarily plant diversity, plant cover and root biomass, were all associated with reduced erosion rates, in line with evidence from salt marsh and terrestrial grasslands (Coops et al. 1996; Pohl et al. 2009; Chen et al. 2012; Liang‐Jun et al. 2013). Interestingly, despite a clear association between plant community type and soil erosion rates, plant diversity indices were consistently selected, above plant community type, as a better predictor variable of soil stability across coastal grassland regions. Abiotic factors, fine‐grained, clay‐rich soils with high soil organic matter were also linked to low erodibility.

The regional model for the erosion‐prone sandy context explained 78% of variation in soil erosion rate with nearly three quarters of that attributed to biotic factors. However, the model for erosion‐resistant clay differed markedly, with less than half (46%) of the statistical variation in erosion rate explained by measured factors. It appears that for clay soils the impact on soil erosion of factors, other than root biomass and soil carbon stock, was much reduced relative to either the combined or erosion‐prone models. Some of the unexplained variation in erosion rate, in Essex soils, may be due to differences in factors not directly measured, such as extracellular polymeric substances (EPS) from micro‐algal communities (Underwood 1997), soil stabilizing organic compounds likely to occur in these regularly inundated salt marsh sediments. For the combined erosion model, further work could include analysis of fine root structure and characteristics, as proposed by Reubens et al. (2007), to identify particular plants or plant communities likely to be important for soil stabilization. Root morphological studies could help further identify the mechanisms behind the plant diversity effects on soil stabilization that were indicated in this study.

## Conclusion

The results presented here clearly indicate that: (1) soil stability is positively associated with plant diversity in salt marsh grasslands; (2) plant species richness is a significant predictor of root biomass; and (3) plant diversity effects are more marked in erosion‐prone than erosion‐resistant soils. Biodiversity–stability effects were partially explained by the positive relationship between plant species richness and root biomass. In addition, where species‐rich plant communities were also functionally diverse, erosion protection could be enhanced by larger root morphological complexity.

## Supporting information


**Appendix S1.** Additional methods section with step‐wise regression predictor selection.

**Appendix S2.** Vegetation core section results.

**Appendix S3.** Supplementary graph of above‐ground and root biomass and soil organic matter.

**Appendix S4.** Additional results section with detailed soil characteristics of Morecambe Bay and Essex salt marsh grasslands.

**Appendix S5.** Supplementary graph of regional differences in erosion rate.
