# Supplementary material for: Soil stabilization linked to plant diversity and environmental context in coastal wetlands
Source: J Veg Sci. 2016 Jan 4;27(2):259–68. doi: 10.1111/jvs.12367 (PMC5111397; doi:10.1111/jvs.12367)
Supplement: Supplementary file 1 — Appendix S1. Additional methods section with step‐wise regression predictor selection. [file JVS-27-259-s001.pdf]

Supporting information to the paper Ford, H *et al*. Soil stabilisation linked to plant diversity and environmental context in coastal wetlands. *Journal of Vegetation Science*. **Appendix S1. Additional methods section with step-wise regression predictor selection.**

Correlations between environmental variables were tested by Spearman's correlation coefficient prior to fitting the step-wise regression model. All bulk density measurements [0 – 10, 10 – 20, 20 – 30 cm, mean (0 – 30 cm depth)] were partially or significantly negatively correlated with the percentage content of clay and silt particles [clay-silt grain size fraction combined (%)] due to the inherent high density of sand particles. Therefore, only 'clay-silt' was used as a potential predictor in step-wise regression and all bulk density measurements were excluded. For sets of related measurements - root biomass (3 depths & total), organic matter (3 depths & mean), carbon stock (3 depths & total) and bulk shear (3 depths & mean) - the variable from each set that explained most variation in erosion rate (highest R^2) was entered into the initial step-wise regression model. This was usually a mean or pooled variable over 0 – 30 cm soil depth. For vegetation variables measured at the quadrat and erosion core level - plant species richness, plant biodiversity (S-W index), plant percentage cover and above ground biomass - the variable from each pair that explained most variation was used in the step-wise regression initial model. In most cases this was quadrat level data.
