# Supplementary material for: Soil stabilization linked to plant diversity and environmental context in coastal wetlands
Source: J Veg Sci. 2016 Jan 4;27(2):259–68. doi: 10.1111/jvs.12367 (PMC5111397; doi:10.1111/jvs.12367)
Supplement: Supplementary file 2 — Appendix S2. Vegetation core section results. [file JVS-27-259-s003.pdf]

Supporting information to the paper Ford, H *et al*. Soil stabilisation linked to plant diversity and environmental context in coastal wetlands. *Journal of Vegetation Science*. **Appendix S2. Vegetation core section results**

Plant species richness and S-W index were both greater in Morecambe Bay than Essex (Table S2), mirroring the quadrat results. Unsurprisingly, core diversity measurements were lower than quadrat diversity measurements across all six sites, reflecting species-area effects. Above-ground biomass was greater in Essex than Morecambe Bay. The mean above-ground biomass estimated from sampling the surface of the core were generally lower than estimates derived from the cut quadrat section, because scarce patches of biomass-rich tussocky plants on average occurred less in the smaller (16 cm diameter) cores than the larger cut sections (25 x 50 cm). The values in table S2 were compared to quadrat level data in their prediction of soil erosion rate. In most cases best predictions were from quadrat level data.

**Table S2.** Mean ± standard deviation species richness, Shannon-Wiener index (S-W) and above-ground biomass of vegetation in six salt marshes of two regions (Essex & Morecambe). Means of sites with different letters (a, b, c) were significantly different. *** signifies significant differences between regions ($p$ < 0.001), ANOVA

| | Essex | | | Morecambe Bay | | | |
|---|---|---|---|---|---|---|---|
| | AH | FW | TM | CS | WP | WS | |
| *Vegetation - core* | | | | | | | |
| Plant species richness | 2.90 ± 1.23 a | 2.68 ± 0.89 a | 1.91 ± 0.75 a | 2.64 ± 1.40 a | 4.5 ± 1.79 b | 4.14 ± 1.13 b | *** |
| S-W index (H') | 0.60 ± 0.37 a | 0.55 ± 0.32 a | 0.34 ± 0.30 a | 0.55 ± 0.45 a | 1.07 ± 0.52 b | 1.05 ± 0.38 b | *** |
| Biomass (kg DW $m^{-2}$) | 0.35 ± 0.22 c | 0.45 ± 0.28 c | 0.27 ± 0.15 bc | 0.03 ± 0.04 a | 0.24 ± 0.28 b | 0.09 ± 0.21 a | *** |

AH = Abbotts Hall, FW = Fingringhoe Wick, TM = Tillingham marsh, CS = Cartmel Sands, WP = West Plain, WS = Warton Sands.
