# Supplementary material for: Soil stabilization linked to plant diversity and environmental context in coastal wetlands
Source: J Veg Sci. 2016 Jan 4;27(2):259–68. doi: 10.1111/jvs.12367 (PMC5111397; doi:10.1111/jvs.12367)
Supplement: Supplementary file 3 — Appendix S3. Supplementary graph of above‐ground and root biomass and soil organic matter. [file JVS-27-259-s004.pdf]

Supporting information to the paper Ford, H *et al*. Soil stabilisation linked to plant diversity and environmental context in coastal wetlands. *Journal of Vegetation Science*. **Appendix S3. Supplementary figure of above-ground and root biomass and soil organic matter**

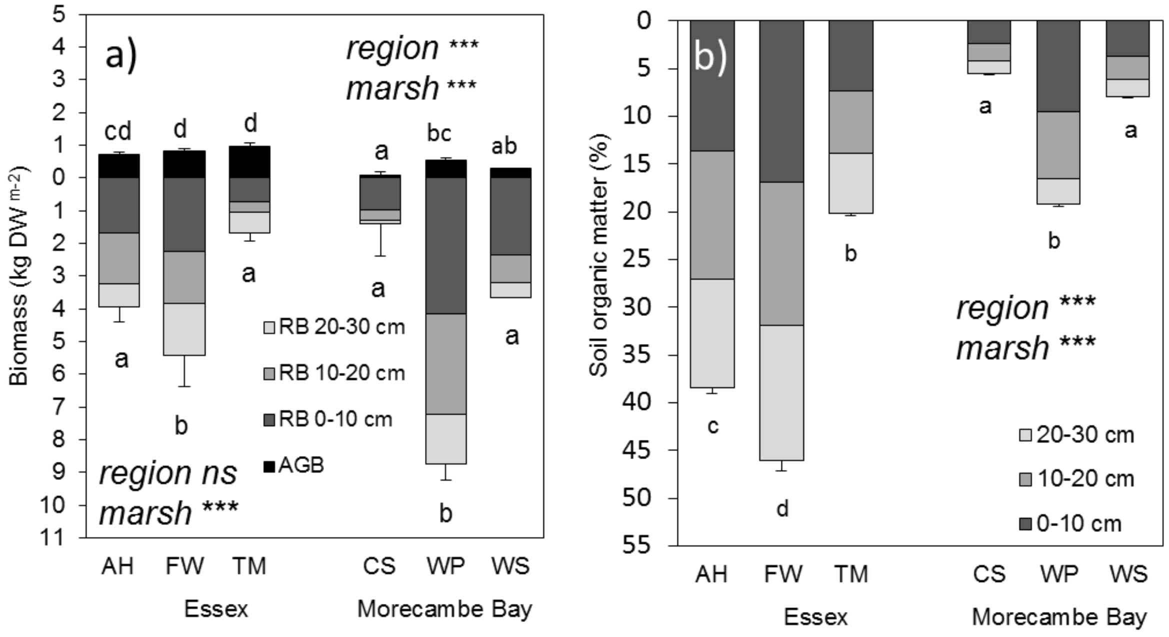

Fig. S3. Illustrating a) above-ground (AGB) and root biomass (RB) and b) soil organic matter content for three depth zones in six saltmarsh sites (AH, FW, TM = Essex; CS, WP, WS = Morecambe Bay). Significant site differences (letters a, b, c, d; $P < 0.05$) and error bars (standard errors) refer to a) AGB and total RB (0-30 cm combined) and b) mean soil organic matter (0-30 cm), not to individual 10 cm sections (shown graphically).
