# Supplementary material for: Soil stabilization linked to plant diversity and environmental context in coastal wetlands
Source: J Veg Sci. 2016 Jan 4;27(2):259–68. doi: 10.1111/jvs.12367 (PMC5111397; doi:10.1111/jvs.12367)
Supplement: Supplementary file 4 — Appendix S4. Additional results section with detailed soil characteristics of Morecambe Bay and Essex salt marsh grasslands. [file JVS-27-259-s005.pdf]

Supporting information to the paper Ford, H *et al*. Soil stabilisation linked to plant diversity and environmental context in coastal wetlands. *Journal of Vegetation Science*. **Appendix S4. Additional results section with detailed soil characteristics of Morecambe Bay and Essex saltmarsh grasslands.**

Elevation relative to MHWN was significantly greater for Morecambe Bay (~ 2.5 m) than Essex (~ 1 m) saltmarsh sites (Table 2) indicating that the Essex saltmarshes were tidally inundated more often (despite differences in tidal range). This was confirmed by soil electrical conductivity, a proxy for salinity, being appreciably greater in Essex (~ 25 mS cm^{-1}; 17 PSU) than Morecambe Bay (~ 4 mS cm^{-1}; 2.5 PSU). Soil moisture content was also greater for Essex (45 – 60 %) than Morecambe Bay (25 – 40 %), although it should be noted that soil erosion tests occurred under conditions of fully saturated soil. Soil pH was significantly greater in Morecambe Bay than Essex, ranging from ~6.5 – 7.5. Bulk density (mean of 0 – 30 cm depth) was significantly greater for Morecambe Bay than for Essex due to the higher density of sand compared to clay or silt. Organic matter content was significantly greater in Essex than Morecambe Bay (Fig. S3) across all soil depths and for mean organic matter (0 – 30 cm depth), but at the site level FW and AH soils were very organic (12 – 15 %), TM and WP moderately so (6 – 7 %) with CS and WS markedly less (1.5 – 3 %). If only organic matter at 0 – 10 cm depth is known, as is common, results from this study indicate that for Essex organic matter content at 10 – 20 cm is ~ 90 – 99 % of the 0 – 10 cm layer, for Morecambe Bay ~ 70 – 80 %. The organic matter content at 20 – 30 cm depth was ~ 85 % of the 0 – 10 zone in Essex, between ~ 30 – 50 % in Morecambe Bay. Soil carbon stock was also greater in Essex (90 - 119 t C ha^{-1}) than Morecambe Bay (CS & WS: 38 & 52 t C ha^{-1}), apart from WP (93 t C ha^{-1}). For sediment grain size, clay-silt fraction was significantly greater in Essex (80 – 90 %) than Morecambe bay (7 – 17 %) saltmarsh sites (Table 1).
