# Supplementary material for: Soil stabilization linked to plant diversity and environmental context in coastal wetlands
Source: J Veg Sci. 2016 Jan 4;27(2):259–68. doi: 10.1111/jvs.12367 (PMC5111397; doi:10.1111/jvs.12367)
Supplement: Supplementary file 5 — Appendix S5. Supplementary graph of regional differences in erosion rate. [file JVS-27-259-s002.pdf]

Supporting information to the paper Ford, H *et al*. Soil stabilisation linked to plant diversity and environmental context in coastal wetlands. *Journal of Vegetation Science*. **Appendix S5. Supplementary graph of regional differences in erosion rate.**

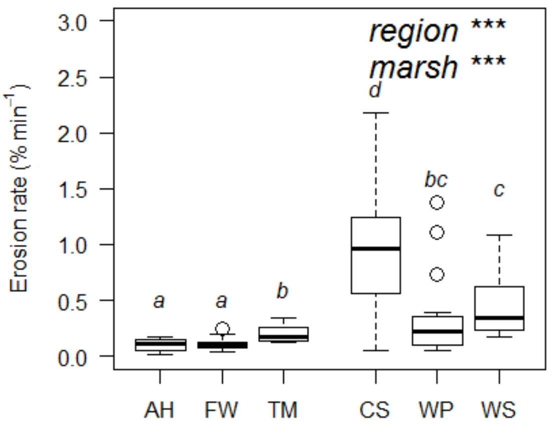

Fig. S5. Soil core erosion rate for six saltmarsh sites (AH, FW, TM = Essex; CS, WP, WS = Morecambe Bay), italicised letters denote significant site differences $P < 0.05$, *** $P < 0.001$. Thick bar = median, box = interquartile range, whiskers = full range, open circles = outliers.
